# Laboratory networking systems to tackle the spread of monkeypox virus – a need of the hour

**DOI:** 10.1097/JS9.0000000000000148

**Published:** 2023-02-16

**Authors:** Vishnu Priya Veeraraghavan, Jyotsna Needamangalam Balaji, Sreenidhi Prakash, Lavina Prashar, Ullas Mony, Krishna Mohan Surapaneni

**Affiliations:** a Centre of Molecular Medicine and Diagnostics (COMManD), Saveetha Dental College and Hospitals, Saveetha Institute of Medical and Technical Sciences, Saveetha University; b Panimalar Medical College Hospital and Research Institute; c SMAART Population Health Informatics Intervention Center, Foundation of Healthcare Technologies Society; d Departments of Biochemistry, Medical Education, Molecular Virology, Research, Clinical Skills and Simulation, Panimalar Medical College Hospital and Research Institute, Varadharajapuram, Chennai, Tamil Nadu, India; e Department of Physiological Sciences, School of Medicine, Unza Ridgeway Campus, University of Zambia, Lusaka, Zambia

*Dear Editor:*

The recent spike of a rare zoonotic infection caused by the monkeypox virus is gaining global attention due to its high transmissibility and fear of stigmatization. Without proper measures to prevent the spread of virus, monkeypox could potentially set the stage for another widespread outbreak leading to worldwide health calamity as witness during the coronavirus disease 2019 pandemic. As a lesson learnt, it is now evident that rapid screening and diagnostic testing is the most efficient approach toward containment of any disease outbreak^1^. We take this opportunity to emphasize on measures to contain the transmission of the virus and possible methods to improve the detection and management of monkeypox disease through improved laboratory networking.

The Centres for Disease Control and Prevention recommend the testing of all patients who present with any noticeable rash resembling monkeypox virus or patients with other symptoms of the disease in order to prevent the recent monkeypox surge from emerging as another pandemic of global concern. Currently, the confirmatory test performed monkeypox virus is the Nucleic Acid Amplification Test that uses the PCR to detect the viral DNA^2^. In view of this, the United States Department of Health and Human Service has expanded the monkeypox testing services by shipping patients’ samples to five major commercial laboratories that includes Aegis Science, Mayo Clinic Laboratories, Sonic Healthcare, Labcorp, and Quest Diagnostics to increase the accessibility and capacity of testing nationwide thereby reducing the cumbersome in local laboratories^3^. Also, the Centres for Disease Control and Prevention ensure that the Laboratory Response Network that was established in collaboration with other public health officials is equipped with diagnostic facilities to detect the monkeypox virus belonging to the genus *Orthopoxvirus*. The Laboratory Response Network was set up with the goal of forming a stable network across the nation to effectively and rapidly share diagnostic tools, interpretation of results, training of laboratory technicians, and reporting of any errors through critical evaluation and communication networks during regular as well as emergency health crisis^4^. Such an intricate laboratory networking system is crucial to distribute highly confidential data and reduce the incidence of faulty procedures and incorrect test results.

In India, the central government has established a network consisting of a total of 15 Virus Research and Diagnostic Laboratories across the country in 13 states to track the prevalence of monkeypox and promote rapid testing facilities^5^. In addition to this, scientists from the ICMR National Institute of Virology in Pune are offering expert training to fellow members from different nations on detection, clinical manifestations, case definitions, collection of samples, handling of samples, and laboratory tool usage to improve their capacity to detect monkeypox cases and screen the suspected population in order to mitigate the transmission of the virus locally and travel-related spread^6^. Despite these initiatives, in a country with 1.4 billion people, 15 network laboratories is certainly suboptimal and requires more vigorous surveillance and diagnostic modalities. In this regard, the Government of India has decided to enhance the monitoring and diagnostic centres in the country at the level of hospitals, entry points, and local communities, along with meticulous contact tracing and screening of suspected individuals^7^.

Throughout the world, monkeypox disease is raising concern, and rapid screening and testing is the most efficient step toward curbing the disease’s outbreak. Not only symptomatic but also asymptomatic patients and close contacts should also be rapidly screened for the monkeypox virus. This mandates a well-organized and rigorous laboratory network system across the world to collect, transport, test, and report the result of the sample. Laboratory networking functions beyond the scope of testing. This complex and vigilant network system serves as a platform for providing emergency response, training of laboratory personnel, communication across different testing centres, surveillance of laboratory data, and diligent management of patient information^8^. Thus, the establishment and maintenance of such robust laboratory networks is highly critical to improving the public health system of a nation or state and is a step toward enhancing the quality of health care service and health data management across the globe.

## Ethical approval

Not applicable.

## Sources of funding

Not funded by any funding agency.

## Author contribution

V.P.V., S.P., J.N.B, L.P., U.M.: conceptualization, data curation, writing – original draft preparation. S.K.M.: conceptualization, study design, data curation, writing – original draft preparation, reviewing and editing, visualization and supervision.

## Conflicts of interest disclosure

No conflict of interest.

## Research registration unique identifying number (UIN)

Not Applicable

## Guarantor

Dr Surapaneni K. Mohan, corresponding author.

## Data statement

This correspondence is based exclusively on resources that are publicly available on the internet and duly cited in the ‘References’ section. No primary data was generated and reported in this manuscript. Therefore, data has not become available to any academic repository.
